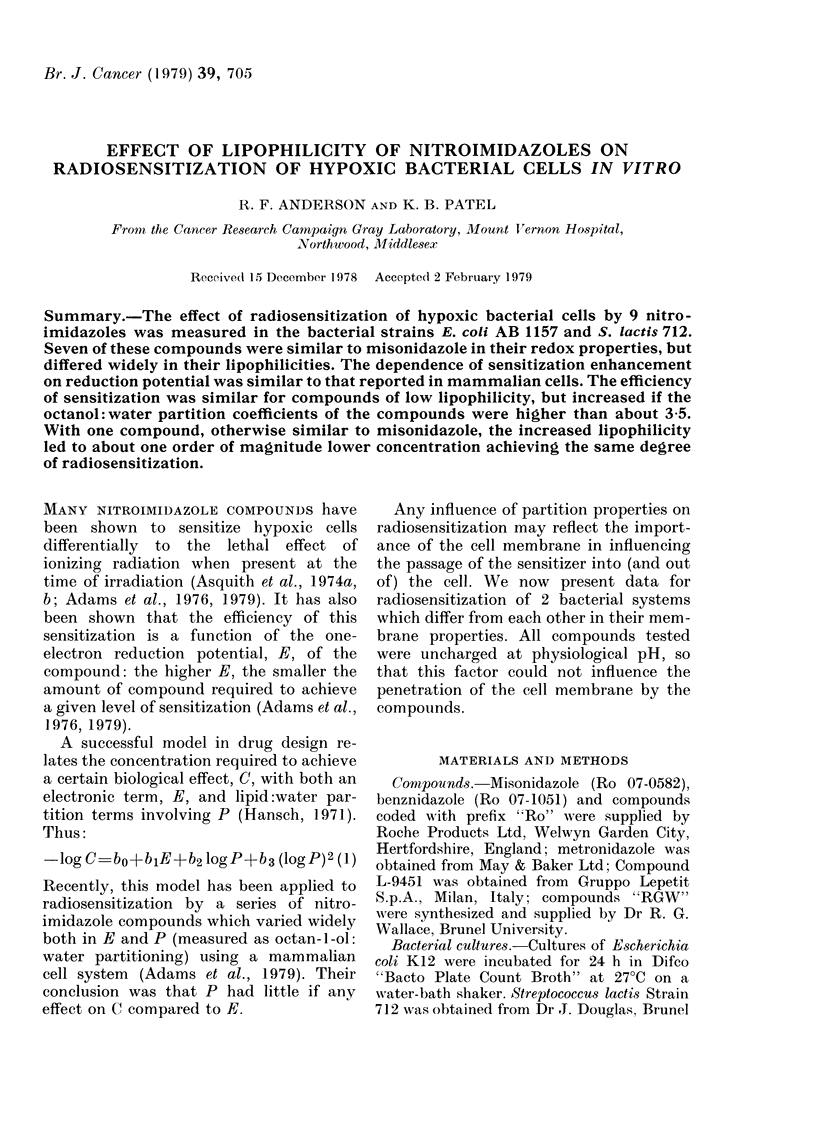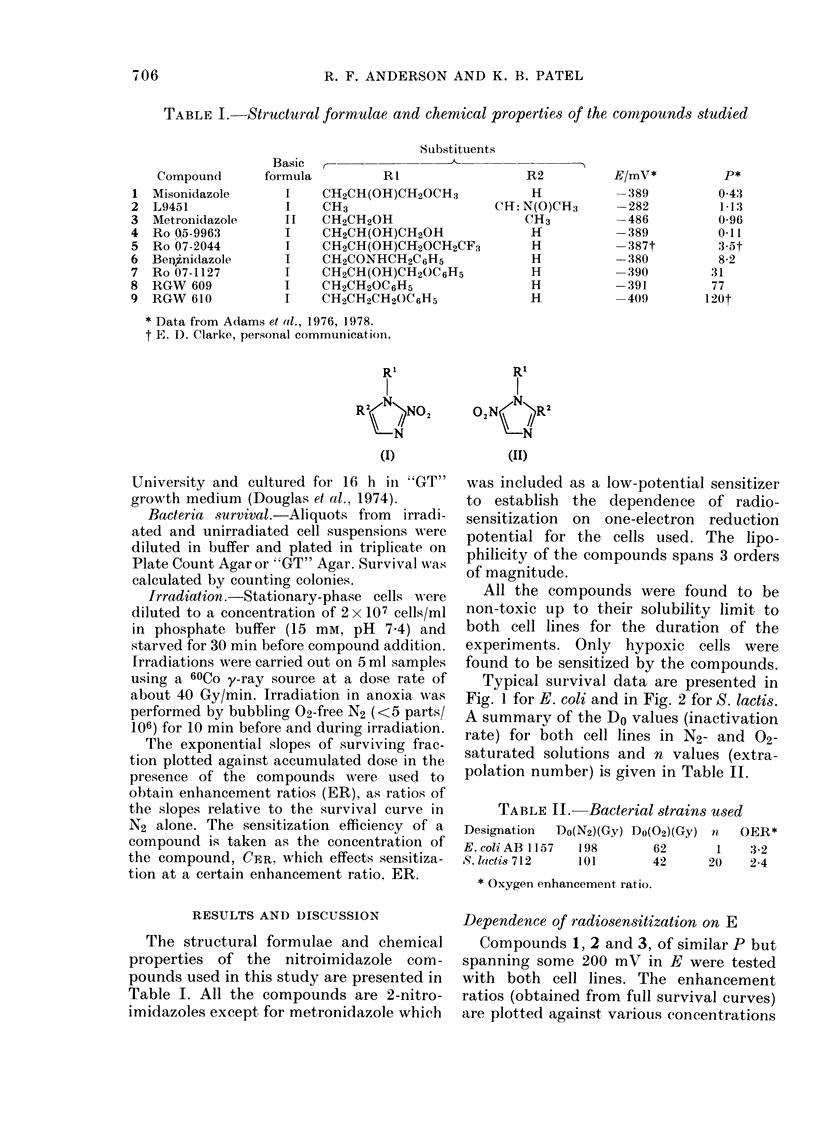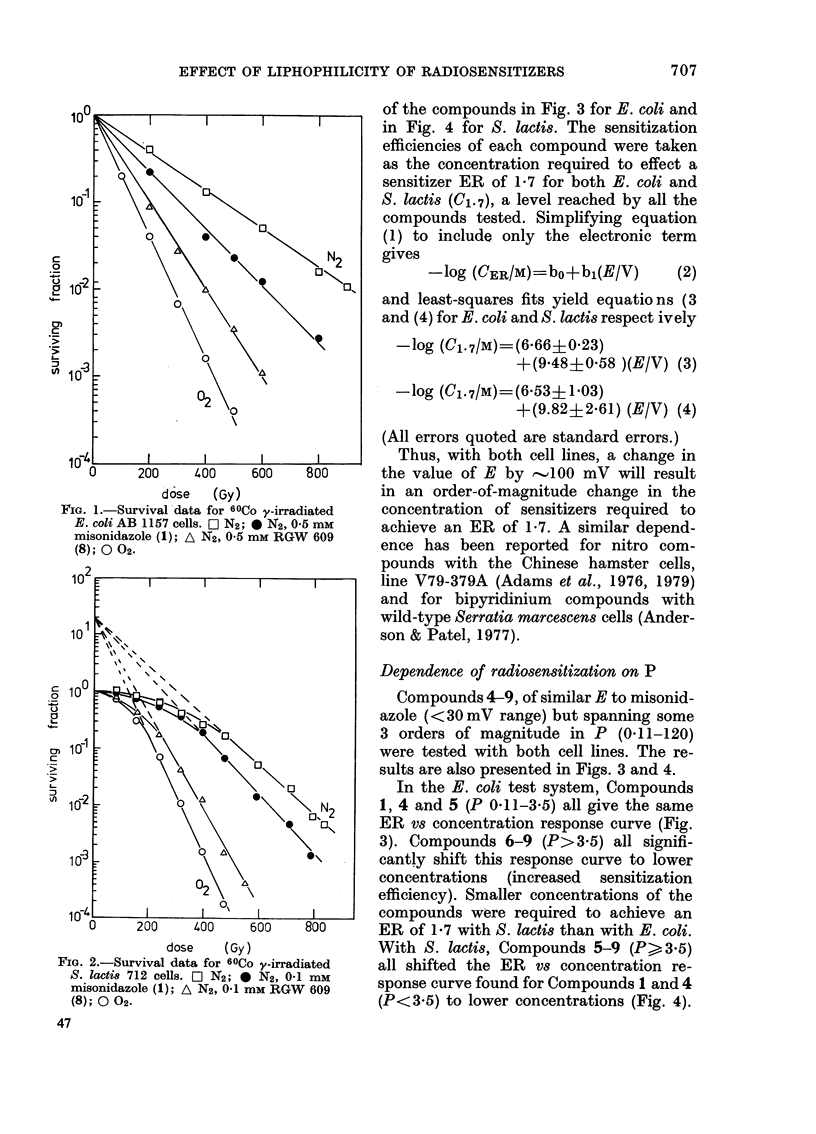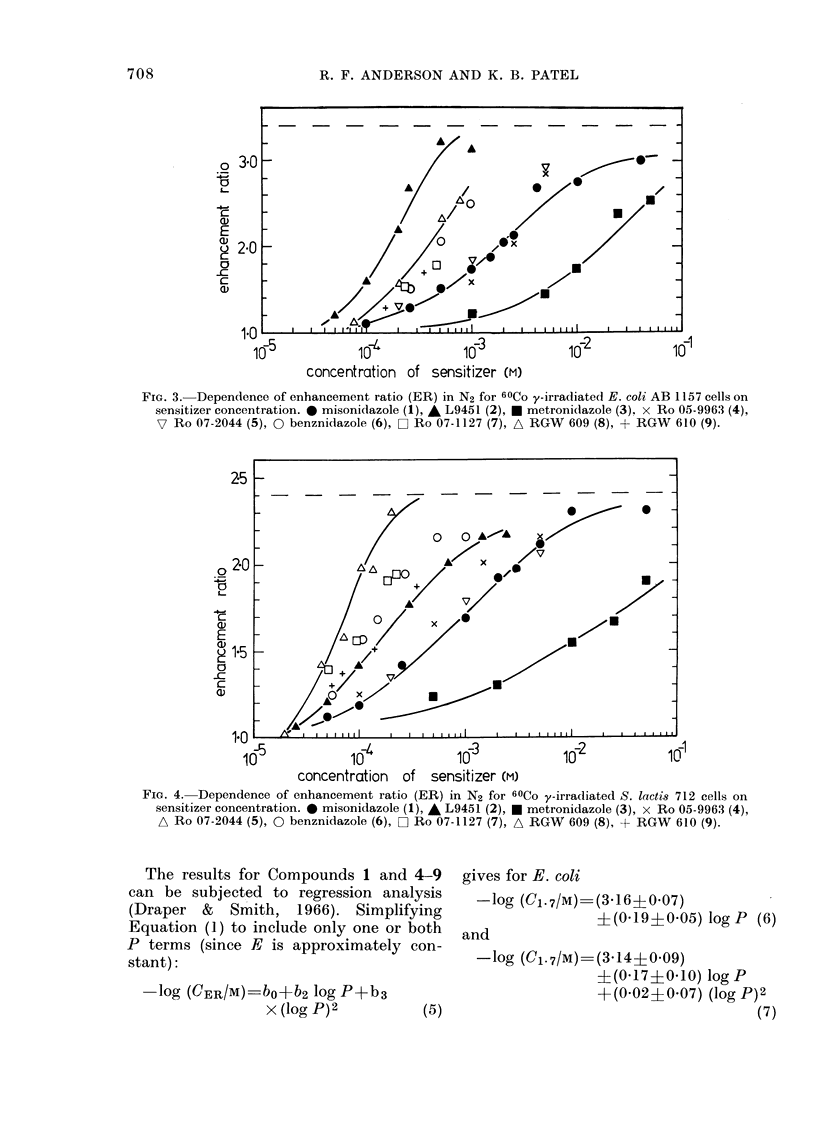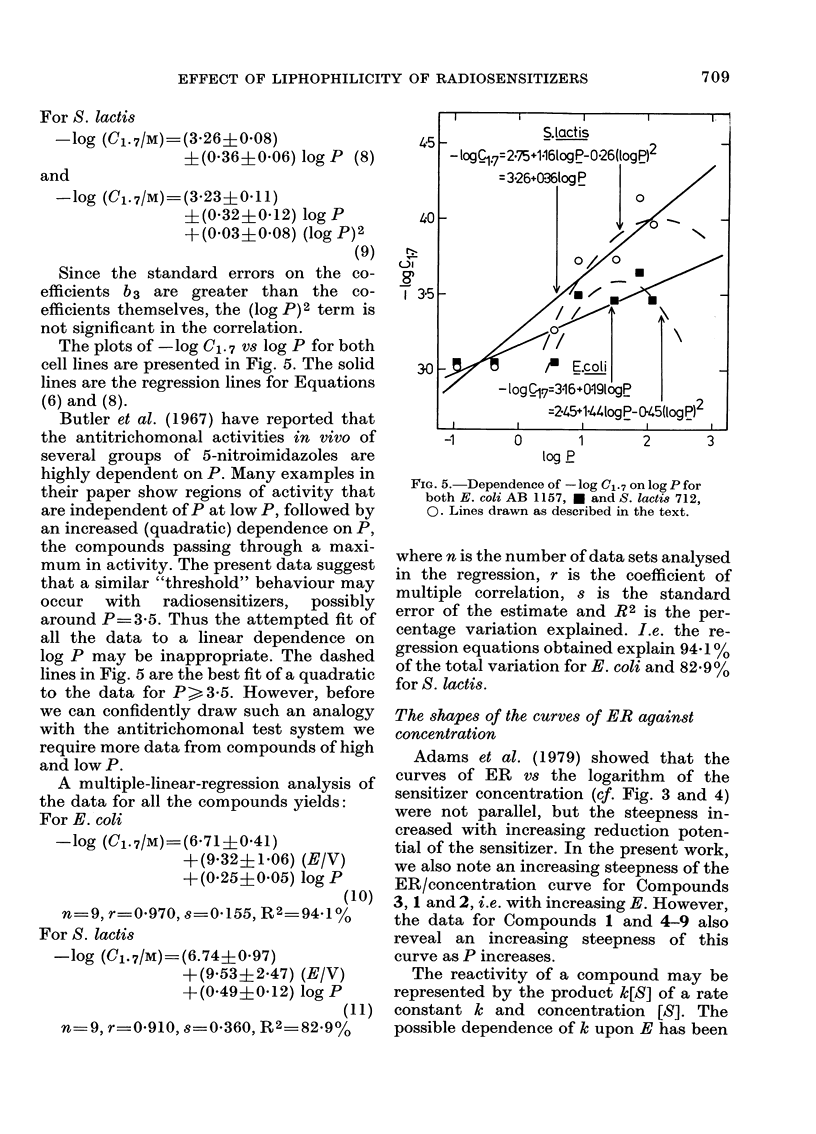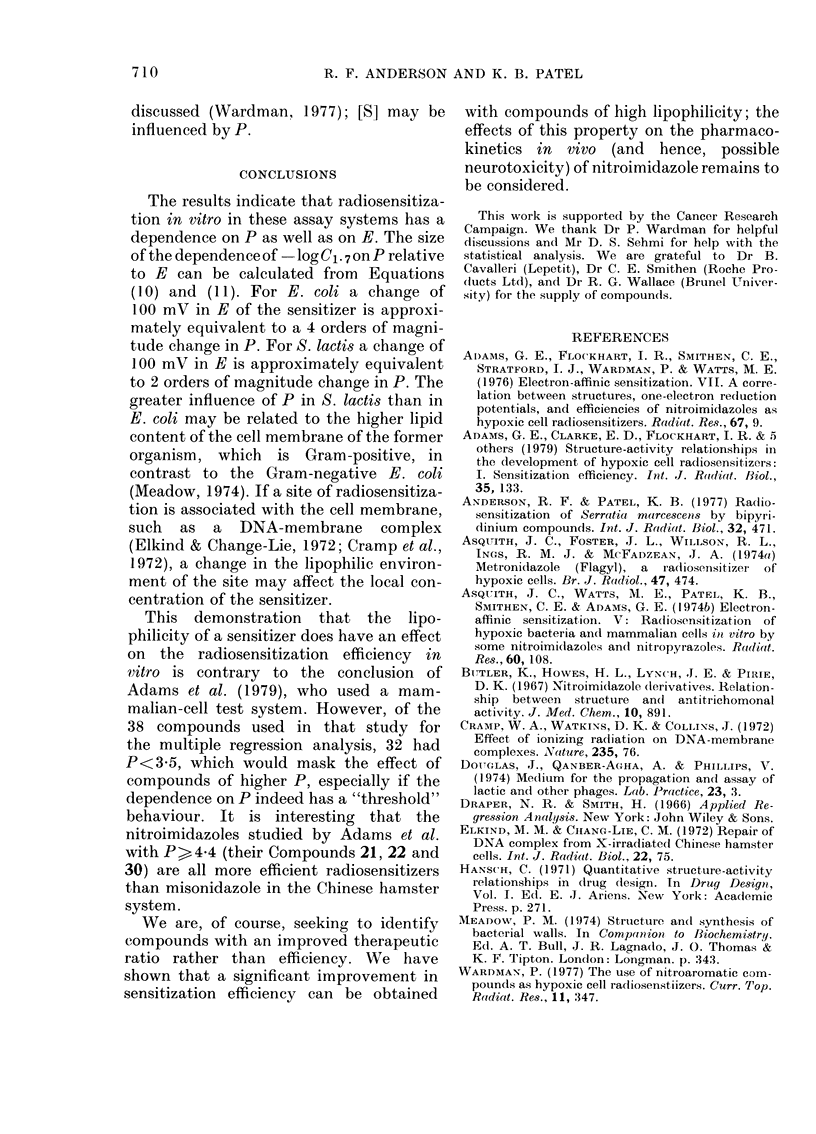# Effect of lipophilicity of nitroimidazoles on radiosensitization of hypoxic bacterial cells in vitro.

**DOI:** 10.1038/bjc.1979.124

**Published:** 1979-06

**Authors:** R. F. Anderson, K. B. Patel

## Abstract

The effect of radiosensitization of hypoxic bacterial cells by 9 nitroimidazoles was measured in the bacterial strains E. coli AB 1157 and S. lactis 712. Seven of these compounds were similar to misonidazole in their redox properties, but differed widely in their lipophilicites. The dependence of sensitization enhancement on reduction potential was similar to that reported in mammalian cells. The efficiency of sensitization was similar for compounds of low lipophilicity, but increased if the octanol: water partition coefficients of the compounds were higher than about 3.5. With one compound, otherwise similar to misonidazole, the increased lipophilicity led to about one order of magnitude lower concentration achieving the same degree of radiosensitization.


Br. J. Cancer (1979) 39, 705

### EFFECT OF LIPOPHILICITY OF NITROIMIDAZOLES ON RADIOSENSITIZATION OF HYPOXIC BACTERIAL CELLS *IN VITRO*

R. F. ANDERSON AND K. B. PATEL

*From the Cancer Research Campaign Gray Laboratory, Mount Vernon Hospital, Northwood, Middlesex*

Received 15 December 1978 Accepted 2 February 1979

**Summary.—The effect of radiosensitization of hypoxic bacterial cells by 9 nitroimidazoles was measured in the bacterial strains *E. coli* AB 1157 and *S. lactis* 712. Seven of these compounds were similar to misonidazole in their redox properties, but differed widely in their lipophilicities. The dependence of sensitization enhancement on reduction potential was similar to that reported in mammalian cells. The efficiency of sensitization was similar for compounds of low lipophilicity, but increased if the octanol: water partition coefficients of the compounds were higher than about 3·5. With one compound, otherwise similar to misonidazole, the increased lipophilicity led to about one order of magnitude lower concentration achieving the same degree of radiosensitization.**

MANY NITROIMIDAZOLE COMPOUNDS have been shown to sensitize hypoxic cells differentially to the lethal effect of ionizing radiation when present at the time of irradiation (Asquith *et al.*, 1974*a*, *b*; Adams *et al.*, 1976, 1979). It has also been shown that the efficiency of this sensitization is a function of the one-electron reduction potential, $E$, of the compound: the higher $E$, the smaller the amount of compound required to achieve a given level of sensitization (Adams *et al.*, 1976, 1979).

A successful model in drug design relates the concentration required to achieve a certain biological effect, $C$, with both an electronic term, $E$, and lipid:water partition terms involving $P$ (Hansch, 1971). Thus:

$$-\log C = b_0 + b_1 E + b_2 \log P + b_3 (\log P)^2 \quad (1)$$

Recently, this model has been applied to radiosensitization by a series of nitroimidazole compounds which varied widely both in $E$ and $P$ (measured as octan-1-ol: water partitioning) using a mammalian cell system (Adams *et al.*, 1979). Their conclusion was that $P$ had little if any effect on C compared to $E$.

Any influence of partition properties on radiosensitization may reflect the importance of the cell membrane in influencing the passage of the sensitizer into (and out of) the cell. We now present data for radiosensitization of 2 bacterial systems which differ from each other in their membrane properties. All compounds tested were uncharged at physiological pH, so that this factor could not influence the penetration of the cell membrane by the compounds.

### MATERIALS AND METHODS

*Compounds*.—Misonidazole (Ro 07-0582), benznidazole (Ro 07-1051) and compounds coded with prefix "Ro" were supplied by Roche Products Ltd, Welwyn Garden City, Hertfordshire, England; metronidazole was obtained from May & Baker Ltd; Compound L-9451 was obtained from Gruppo Lepetit S.p.A., Milan, Italy; compounds "RGW" were synthesized and supplied by Dr R. G. Wallace, Brunel University.

*Bacterial cultures*.—Cultures of *Escherichia coli* K12 were incubated for 24 h in Difco "Bacto Plate Count Broth" at 27°C on a water-bath shaker. *Streptococcus lactis* Strain 712 was obtained from Dr J. Douglas, Brunel

TABLE I.—*Structural formulae and chemical properties of the compounds studied*

| | Compound | Basic formula | Substituents R1 | R2 | *E*/mV* | *P** |
|---|---|---|---|---|---|---|
| 1 | Misonidazole | I | $CH_2CH(OH)CH_2OCH_3$ | H | −389 | 0·43 |
| 2 | L9451 | I | $CH_3$ | $CH{:}N(O)CH_3$ | −282 | 1·13 |
| 3 | Metronidazole | II | $CH_2CH_2OH$ | $CH_3$ | −486 | 0·96 |
| 4 | Ro 05-9963 | I | $CH_2CH(OH)CH_2OH$ | H | −389 | 0·11 |
| 5 | Ro 07-2044 | I | $CH_2CH(OH)CH_2OCH_2CF_3$ | H | −387† | 3·5† |
| 6 | Benznidazole | I | $CH_2CONHCH_2C_6H_5$ | H | −380 | 8·2 |
| 7 | Ro 07-1127 | I | $CH_2CH(OH)CH_2OC_6H_5$ | H | −390 | 31 |
| 8 | RGW 609 | I | $CH_2CH_2OC_6H_5$ | H | −391 | 77 |
| 9 | RGW 610 | I | $CH_2CH_2CH_2OC_6H_5$ | H | −409 | 120† |

* Data from Adams *et al.*, 1976, 1978.
† E. D. Clarke, personal communication.

(I)

(II)

University and cultured for 16 h in "GT" growth medium (Douglas *et al.*, 1974).

*Bacteria survival.*—Aliquots from irradiated and unirradiated cell suspensions were diluted in buffer and plated in triplicate on Plate Count Agar or "GT" Agar. Survival was calculated by counting colonies.

*Irradiation.*—Stationary-phase cells were diluted to a concentration of $2 \times 10^7$ cells/ml in phosphate buffer (15 mM, pH 7·4) and starved for 30 min before compound addition. Irradiations were carried out on 5 ml samples using a $^{60}Co$ $\gamma$-ray source at a dose rate of about 40 Gy/min. Irradiation in anoxia was performed by bubbling $O_2$-free $N_2$ (<5 parts/$10^6$) for 10 min before and during irradiation.

The exponential slopes of surviving fraction plotted against accumulated dose in the presence of the compounds were used to obtain enhancement ratios (ER), as ratios of the slopes relative to the survival curve in $N_2$ alone. The sensitization efficiency of a compound is taken as the concentration of the compound, $C_{ER}$, which effects sensitization at a certain enhancement ratio, ER.

### RESULTS AND DISCUSSION

The structural formulae and chemical properties of the nitroimidazole compounds used in this study are presented in Table I. All the compounds are 2-nitroimidazoles except for metronidazole which was included as a low-potential sensitizer to establish the dependence of radiosensitization on one-electron reduction potential for the cells used. The lipophilicity of the compounds spans 3 orders of magnitude.

All the compounds were found to be non-toxic up to their solubility limit to both cell lines for the duration of the experiments. Only hypoxic cells were found to be sensitized by the compounds.

Typical survival data are presented in Fig. 1 for *E. coli* and in Fig. 2 for *S. lactis*. A summary of the $D_0$ values (inactivation rate) for both cell lines in $N_2$- and $O_2$-saturated solutions and *n* values (extrapolation number) is given in Table II.

TABLE II.—*Bacterial strains used*

| Designation | $D_0(N_2)$(Gy) | $D_0(O_2)$(Gy) | *n* | OER* |
|---|---|---|---|---|
| *E. coli* AB 1157 | 198 | 62 | 1 | 3·2 |
| *S. lactis* 712 | 101 | 42 | 20 | 2·4 |

* Oxygen enhancement ratio.

### *Dependence of radiosensitization on* E

Compounds **1**, **2** and **3**, of similar *P* but spanning some 200 mV in *E* were tested with both cell lines. The enhancement ratios (obtained from full survival curves) are plotted against various concentrations

FIG. 1.—Survival data for $^{60}Co$ $\gamma$-irradiated *E. coli* AB 1157 cells. □ $N_2$; ● $N_2$, 0·5 mM misonidazole (**1**); △ $N_2$, 0·5 mM RGW 609 (**8**); ○ $O_2$.

FIG. 2.—Survival data for $^{60}Co$ $\gamma$-irradiated *S. lactis* 712 cells. □ $N_2$; ● $N_2$, 0·1 mM misonidazole (**1**); △ $N_2$, 0·1 mM RGW 609 (**8**); ○ $O_2$.

of the compounds in Fig. 3 for *E. coli* and in Fig. 4 for *S. lactis*. The sensitization efficiencies of each compound were taken as the concentration required to effect a sensitizer ER of 1·7 for both *E. coli* and *S. lactis* ($C_{1\cdot 7}$), a level reached by all the compounds tested. Simplifying equation (1) to include only the electronic term gives

$$-\log (C_{ER}/\text{M}) = b_0 + b_1(E/V) \quad (2)$$

and least-squares fits yield equations (3 and (4) for *E. coli* and *S. lactis* respectively

$$-\log (C_{1\cdot 7}/\text{M}) = (6\cdot 66 \pm 0\cdot 23) + (9\cdot 48 \pm 0\cdot 58)(E/V) \quad (3)$$

$$-\log (C_{1\cdot 7}/\text{M}) = (6\cdot 53 \pm 1\cdot 03) + (9.82 \pm 2\cdot 61)(E/V) \quad (4)$$

(All errors quoted are standard errors.)

Thus, with both cell lines, a change in the value of $E$ by ~100 mV will result in an order-of-magnitude change in the concentration of sensitizers required to achieve an ER of 1·7. A similar dependence has been reported for nitro compounds with the Chinese hamster cells, line V79-379A (Adams *et al.*, 1976, 1979) and for bipyridinium compounds with wild-type *Serratia marcescens* cells (Anderson & Patel, 1977).

*Dependence of radiosensitization on* P

Compounds **4–9**, of similar $E$ to misonidazole (<30 mV range) but spanning some 3 orders of magnitude in $P$ (0·11–120) were tested with both cell lines. The results are also presented in Figs. 3 and 4.

In the *E. coli* test system, Compounds **1**, **4** and **5** ($P$ 0·11–3·5) all give the same ER *vs* concentration response curve (Fig. 3). Compounds **6–9** ($P>3\cdot 5$) all significantly shift this response curve to lower concentrations (increased sensitization efficiency). Smaller concentrations of the compounds were required to achieve an ER of 1·7 with *S. lactis* than with *E. coli*. With *S. lactis*, Compounds **5–9** ($P \geqslant 3\cdot 5$) all shifted the ER *vs* concentration response curve found for Compounds **1** and **4** ($P<3\cdot 5$) to lower concentrations (Fig. 4).

FIG. 3.—Dependence of enhancement ratio (ER) in $N_2$ for $^{60}Co$ $\gamma$-irradiated *E. coli* AB 1157 cells on sensitizer concentration. ● misonidazole (**1**), ▲ L9451 (**2**), ■ metronidazole (**3**), × Ro 05-9963 (**4**), ▽ Ro 07-2044 (**5**), ○ benznidazole (**6**), □ Ro 07-1127 (**7**), △ RGW 609 (**8**), + RGW 610 (**9**).

FIG. 4.—Dependence of enhancement ratio (ER) in $N_2$ for $^{60}Co$ $\gamma$-irradiated *S. lactis* 712 cells on sensitizer concentration. ● misonidazole (**1**), ▲ L9451 (**2**), ■ metronidazole (**3**), × Ro 05-9963 (**4**), △ Ro 07-2044 (**5**), ○ benznidazole (**6**), □ Ro 07-1127 (**7**), △ RGW 609 (**8**), + RGW 610 (**9**).

The results for Compounds **1** and **4–9** can be subjected to regression analysis (Draper & Smith, 1966). Simplifying Equation (1) to include only one or both $P$ terms (since $E$ is approximately constant):

$$-\log (C_{ER}/\text{M}) = b_0 + b_2 \log P + b_3 \times (\log P)^2 \quad (5)$$

gives for *E. coli*

$$-\log (C_{1\cdot7}/\text{M}) = (3{\cdot}16 \pm 0{\cdot}07) \pm (0{\cdot}19 \pm 0{\cdot}05) \log P \quad (6)$$

and

$$-\log (C_{1\cdot7}/\text{M}) = (3{\cdot}14 \pm 0{\cdot}09) \pm (0{\cdot}17 \pm 0{\cdot}10) \log P + (0{\cdot}02 \pm 0{\cdot}07) (\log P)^2 \quad (7)$$

For *S. lactis*

$$-\log (C_{1\cdot 7}/\text{M}) = (3\cdot 26 \pm 0\cdot 08) \pm (0\cdot 36 \pm 0\cdot 06) \log P \quad (8)$$

and

$$-\log (C_{1\cdot 7}/\text{M}) = (3\cdot 23 \pm 0\cdot 11) \pm (0\cdot 32 \pm 0\cdot 12) \log P + (0\cdot 03 \pm 0\cdot 08) (\log P)^2 \quad (9)$$

Since the standard errors on the coefficients $b_3$ are greater than the coefficients themselves, the $(\log P)^2$ term is not significant in the correlation.

The plots of $-\log C_{1\cdot 7}$ *vs* $\log P$ for both cell lines are presented in Fig. 5. The solid lines are the regression lines for Equations (6) and (8).

Butler *et al.* (1967) have reported that the antitrichomonal activities *in vivo* of several groups of 5-nitroimidazoles are highly dependent on $P$. Many examples in their paper show regions of activity that are independent of $P$ at low $P$, followed by an increased (quadratic) dependence on $P$, the compounds passing through a maximum in activity. The present data suggest that a similar "threshold" behaviour may occur with radiosensitizers, possibly around $P = 3\cdot 5$. Thus the attempted fit of all the data to a linear dependence on $\log P$ may be inappropriate. The dashed lines in Fig. 5 are the best fit of a quadratic to the data for $P \geqslant 3\cdot 5$. However, before we can confidently draw such an analogy with the antitrichomonal test system we require more data from compounds of high and low $P$.

A multiple-linear-regression analysis of the data for all the compounds yields:

For *E. coli*

$$-\log (C_{1\cdot 7}/\text{M}) = (6\cdot 71 \pm 0\cdot 41) + (9\cdot 32 \pm 1\cdot 06) (E/\text{V}) + (0\cdot 25 \pm 0\cdot 05) \log P \quad (10)$$

$n = 9$, $r = 0\cdot 970$, $s = 0\cdot 155$, $R^2 = 94\cdot 1\%$

For *S. lactis*

$$-\log (C_{1\cdot 7}/\text{M}) = (6.74 \pm 0\cdot 97) + (9\cdot 53 \pm 2\cdot 47) (E/\text{V}) + (0\cdot 49 \pm 0\cdot 12) \log P \quad (11)$$

$n = 9$, $r = 0\cdot 910$, $s = 0\cdot 360$, $R^2 = 82\cdot 9\%$

FIG. 5.—Dependence of $-\log C_{1\cdot 7}$ on $\log P$ for both *E. coli* AB 1157, ■ and *S. lactis* 712, ○. Lines drawn as described in the text.

where $n$ is the number of data sets analysed in the regression, $r$ is the coefficient of multiple correlation, $s$ is the standard error of the estimate and $R^2$ is the percentage variation explained. *I.e.* the regression equations obtained explain 94·1% of the total variation for *E. coli* and 82·9% for *S. lactis*.

*The shapes of the curves of ER against concentration*

Adams *et al.* (1979) showed that the curves of ER *vs* the logarithm of the sensitizer concentration (*cf.* Fig. 3 and 4) were not parallel, but the steepness increased with increasing reduction potential of the sensitizer. In the present work, we also note an increasing steepness of the ER/concentration curve for Compounds **3**, **1** and **2**, *i.e.* with increasing $E$. However, the data for Compounds **1** and **4**–**9** also reveal an increasing steepness of this curve as $P$ increases.

The reactivity of a compound may be represented by the product $k[S]$ of a rate constant $k$ and concentration $[S]$. The possible dependence of $k$ upon $E$ has been discussed (Wardman, 1977); [S] may be influenced by $P$.

### CONCLUSIONS

The results indicate that radiosensitization *in vitro* in these assay systems has a dependence on $P$ as well as on $E$. The size of the dependence of $-\log C_{1\cdot 7}$ on $P$ relative to $E$ can be calculated from Equations (10) and (11). For *E. coli* a change of 100 mV in $E$ of the sensitizer is approximately equivalent to a 4 orders of magnitude change in $P$. For *S. lactis* a change of 100 mV in $E$ is approximately equivalent to 2 orders of magnitude change in $P$. The greater influence of $P$ in *S. lactis* than in *E. coli* may be related to the higher lipid content of the cell membrane of the former organism, which is Gram-positive, in contrast to the Gram-negative *E. coli* (Meadow, 1974). If a site of radiosensitization is associated with the cell membrane, such as a DNA-membrane complex (Elkind & Change-Lie, 1972; Cramp *et al.*, 1972), a change in the lipophilic environment of the site may affect the local concentration of the sensitizer.

This demonstration that the lipophilicity of a sensitizer does have an effect on the radiosensitization efficiency *in vitro* is contrary to the conclusion of Adams *et al.* (1979), who used a mammalian-cell test system. However, of the 38 compounds used in that study for the multiple regression analysis, 32 had $P<3{\cdot}5$, which would mask the effect of compounds of higher $P$, especially if the dependence on $P$ indeed has a "threshold" behaviour. It is interesting that the nitroimidazoles studied by Adams *et al.* with $P \geqslant 4{\cdot}4$ (their Compounds **21**, **22** and **30**) are all more efficient radiosensitizers than misonidazole in the Chinese hamster system.

We are, of course, seeking to identify compounds with an improved therapeutic ratio rather than efficiency. We have shown that a significant improvement in sensitization efficiency can be obtained with compounds of high lipophilicity; the effects of this property on the pharmacokinetics *in vivo* (and hence, possible neurotoxicity) of nitroimidazole remains to be considered.

This work is supported by the Cancer Research Campaign. We thank Dr P. Wardman for helpful discussions and Mr D. S. Sehmi for help with the statistical analysis. We are grateful to Dr B. Cavalleri (Lepetit), Dr C. E. Smithen (Roche Products Ltd), and Dr R. G. Wallace (Brunel University) for the supply of compounds.

### REFERENCES

Adams, G. E., Flockhart, I. R., Smithen, C. E., Stratford, I. J., Wardman, P. & Watts, M. E. (1976) Electron-affinic sensitization. VII. A correlation between structures, one-electron reduction potentials, and efficiencies of nitroimidazoles as hypoxic cell radiosensitizers. *Radiat. Res.*, **67**, 9.

Adams, G. E., Clarke, E. D., Flockhart, I. R. & 5 others (1979) Structure-activity relationships in the development of hypoxic cell radiosensitizers: I. Sensitization efficiency. *Int. J. Radiat. Biol.*, **35**, 133.

Anderson, R. F. & Patel, K. B. (1977) Radiosensitization of *Serratia marcescens* by bipyridinium compounds. *Int. J. Radiat. Biol.*, **32**, 471.

Asquith, J. C., Foster, J. L., Willson, R. L., Ings, R. M. J. & McFadzean, J. A. (1974*a*) Metronidazole (Flagyl), a radiosensitizer of hypoxic cells. *Br. J. Radiol.*, **47**, 474.

Asquith, J. C., Watts, M. E., Patel, K. B., Smithen, C. E. & Adams, G. E. (1974*b*) Electron-affinic sensitization. V: Radiosensitization of hypoxic bacteria and mammalian cells *in vitro* by some nitroimidazoles and nitropyrazoles. *Radiat. Res.*, **60**, 108.

Butler, K., Howes, H. L., Lynch, J. E. & Pirie, D. K. (1967) Nitroimidazole derivatives. Relationship between structure and antitrichomonal activity. *J. Med. Chem.*, **10**, 891.

Cramp, W. A., Watkins, D. K. & Collins, J. (1972) Effect of ionizing radiation on DNA-membrane complexes. *Nature*, **235**, 76.

Douglas, J., Qanber-Agha, A. & Phillips, V. (1974) Medium for the propagation and assay of lactic and other phages. *Lab. Practice*, **23**, 3.

Draper, N. R. & Smith, H. (1966) *Applied Regression Analysis*. New York: John Wiley & Sons.

Elkind, M. M. & Chang-Lie, C. M. (1972) Repair of DNA complex from X-irradiated Chinese hamster cells. *Int. J. Radiat. Biol.*, **22**, 75.

Hansch, C. (1971) Quantitative structure-activity relationships in drug design. In *Drug Design*, Vol. I. Ed. E. J. Ariens. New York: Academic Press. p. 271.

Meadow, P. M. (1974) Structure and synthesis of bacterial walls. In *Companion to Biochemistry*. Ed. A. T. Bull, J. R. Lagnado, J. O. Thomas & K. F. Tipton. London: Longman. p. 343.

Wardman, P. (1977) The use of nitroaromatic compounds as hypoxic cell radiosenstiizers. *Curr. Top. Radiat. Res.*, **11**, 347.